# One-Pot Radiosynthesis and Biological Evaluation of a Caspase-3 Selective 5-[^123,125^I]iodo-1,2,3-triazole derived Isatin SPECT Tracer

**DOI:** 10.1038/s41598-019-55992-0

**Published:** 2019-12-17

**Authors:** Matthias Glaser, Vineeth Rajkumar, Seckou Diocou, Thibault Gendron, Ran Yan, Pak Kwan Brian Sin, Kerstin Sander, Laurence Carroll, R. Barbara Pedley, Eric O. Aboagye, Timothy H. Witney, Erik Årstad

**Affiliations:** 1 Centre for Radiopharmaceutical Chemistry, University College London, 5 Gower Place, London, WC1E 6BS United Kingdom; 2 Department of Chemistry, University College London, 20 Gordon Street, London, WC1H 0AJ United Kingdom; 3 UCL, Cancer Institute, 72 Huntley Street, London, WC1E 6DD UK; 4 King’s College London, School of Biomedical Engineering and Imaging Sciences, St. Thomas’ Hospital, SE1 7EH London, United Kingdom; 5 Imperial College London, Science, Technology & Medicine, Department of Medicine, Hammersmith Hospital, DuCane Road, London, W12 0NN United Kingdom; 6 Centre for Advanced Biomedical Imaging, Division of Medicine, University College London, London, United Kingdom

**Keywords:** Cancer, Chemical tools

## Abstract

Induction of apoptosis is often necessary for successful cancer therapy, and the non-invasive monitoring of apoptosis post-therapy could assist in clinical decision making. Isatins are a class of compounds that target activated caspase-3 during apoptosis. Here we report the synthesis of the 5-iodo-1,2,3-triazole (FITI) analog of the PET tracer [^18^F]ICMT11 as a candidate tracer for imaging of apoptosis with SPECT, as well as PET. Labelling with radioiodine (^123,125^I) was achieved in 55 ± 12% radiochemical yield through a chelator-accelerated one-pot cycloaddition reaction mediated by copper(I) catalysis. The caspase-3 binding affinity and selectivity of FITI compares favourably to that of [^18^F]ICMT11 (K_i_ = 6.1 ± 0.9 nM and 12.4 ± 4.7 nM, respectively). In biodistribution studies, etoposide-induced cell death in a SW1222 xenograft model resulted in a 2-fold increase in tumour uptake of the tracer. However, the tumour uptake was too low to allow *in vivo* imaging of apoptosis with SPECT.

## Introduction

Programmed cell death, also known as apoptosis, is a highly regulated biochemical process, required for tissue homeostasis, growth and embryonic development^1,2^. Aberrant regulation of apoptotic cell death is a hallmark of a range of pathologies – from cancer, ischemia (stroke, myocardial infarction), autoimmunity, inflammation and neuro-degeneration, to allograft rejection and pathogenic infections^3^. In addition to (patho)physiological roles, apoptosis, necrosis or other recently described forms of cell death, such as necroptosis^4^, are the surrogate endpoint for most anticancer therapies^5^. Current methods used in the clinic to measure treatment efficacy rely on measuring changes in tumour size under the guidelines of the Response Evaluation Criteria in Solid tumours^6^. This approach, however, lacks sensitivity, and many weeks may elapse before there is evidence of tumour volume shrinkage. There is therefore a need to develop novel methods to non-invasively assess apoptotic cell death early after the initiation of therapy^7^. The use of molecular imaging techniques, such as positron emission tomography (PET), and dynamic nuclear polarisation, has shown promise for monitoring of drug-induced cell death^8,9^. Early radiolabelling studies concentrated on the measurement of cell membrane changes, such as the exposure of phosphatidylserine (PS) on the outer leaflet of the plasma membrane using annexin-V, a small 56 kDa protein^10,11^. The poor pharmacokinetic profile of annexin V in humans, along with non-specific retention in viable tumour tissue has, however, precluded further clinical development of this radiotracer in its current form^12^.

**Figure 1:**
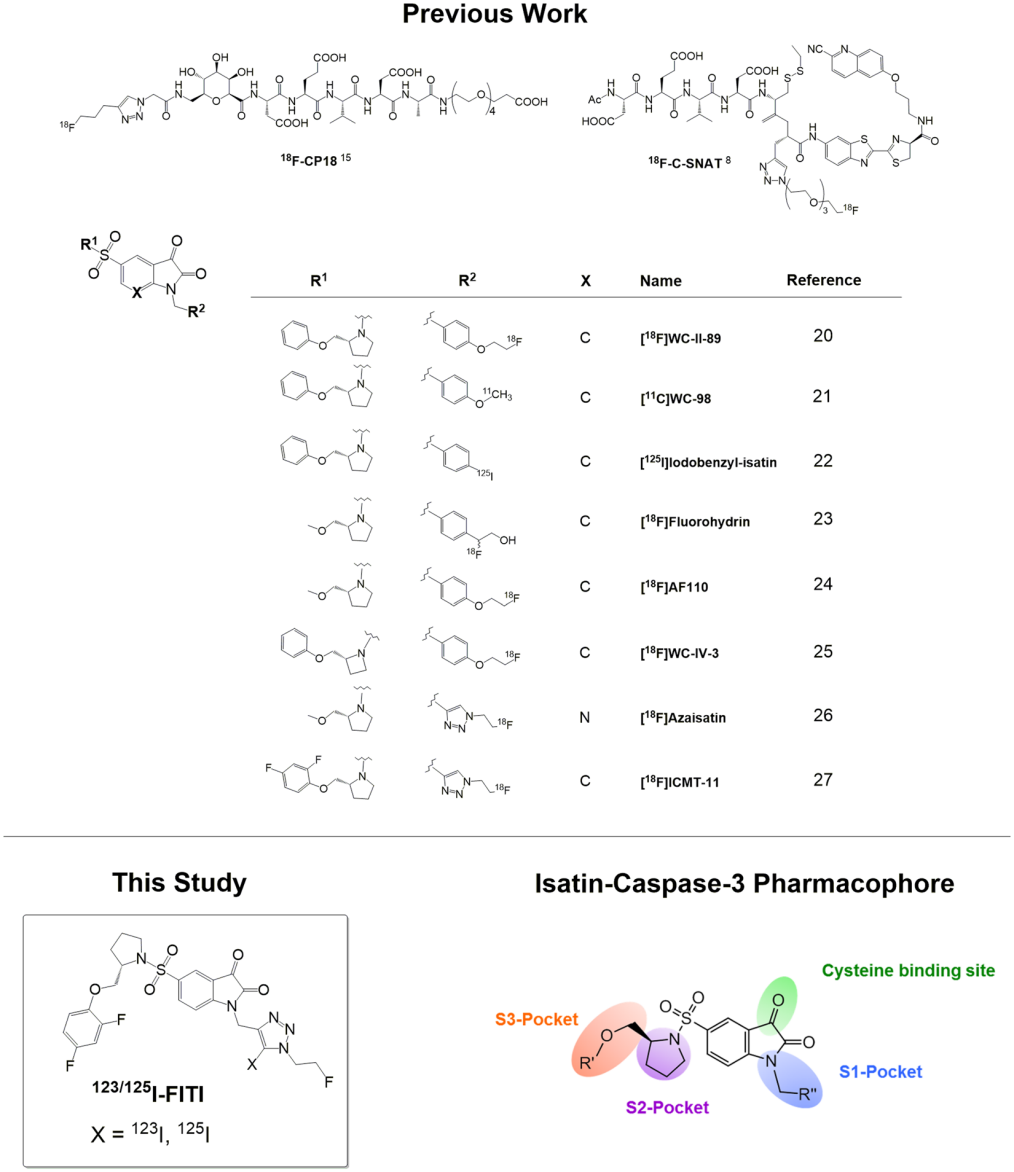
Structures of selected caspase-3 binding PET radiotracers and pharmacophore model for binding interactions of isatins with caspase 3^34^.

The current mechanistic model of apoptosis distinguishes between two converging extrinsic and intrinsic signalling pathways, *via* membrane death receptors and mitochondria, respectively^13^. Caspases (cysteine aspartyl-specific proteases) are essential for the intracellular transmission of the apoptotic signal. These enzymes can be classified either as initiators (caspases-2, -8, and -10), or effectors (caspases-3, -6, and -7)^14^. Due to its central role in the execution of the apoptotic pathway, it is caspase-3 expression that draws the main interest as a marker for targeted molecular imaging techniques^7,15–17^. Various caspase-3 specific peptide tracers such as ^18^F-CP18^18,19^, or ^18^F-C-SNAT^8^ have been described. The discovery of a non-peptidic caspase-3 inhibiting isatin analog has given rise to numerous radiolabelled tracers based on the isatin structural motif^15^. Figure 1 provides an overview of previously reported peptidic and isatin derived caspase-3 radiotracers^20–27^. The isatin derived radiotracer ^18^F-ICMT-11 has shown particularly encouraging results in preclinical studies^17,27–32^, and is the first of this class of tracers to have progressed to first-in-man imaging studies^31^.

According to the caspase-3 pharmacophore model (Fig. 1), the pyrrolidine sulfonamide moiety of isatin analogues is crucial for binding in the S2 hydrophobic pocket^33^, while interactions of *N*-alkylated substituents with the S1 domain also contribute to binding affinity^34^. The S1 binding domain was originally proposed to be a lipophilic pocket, due to the high affinities observed for isatin-derived ligands decorated with *N*-benzylic substituents. Subsequent studies revealed that 1,2,3-triazoles also provide favourable interactions with the S1 binding domain, as demonstrated by the high caspase-3 affinity observed for [^18^F]ICMT-11^27^. This suggests that the S1 binding pocket encompasses both polar and lipophilic domains. We therefore speculated that addition of an iodine substituent to the triazole ring of ICMT-11 could further enhance the affinity by allowing for broader interactions within the S1 binding pocket. Motivated by the prospect of developing a high affinity caspase-3 selective SPECT tracer, herein we developed the 5-iodo-1,2,3-triazole derivative of ICMT-11. Furthermore, we also report the synthesis of ^123/125^I-labelled analogues and their biological evaluation.

## Results and Discussion

### Synthesis and radiolabelling

**Figure 2:**
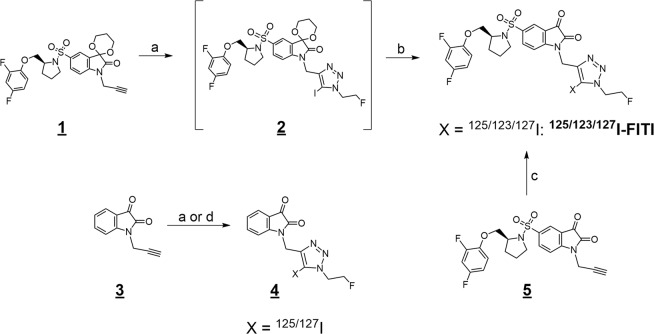
Reaction schemes. Synthesis of [^**123/125/127**^I]**FITI** and the negative control compound **4**. Reaction conditions: (**a**) 2-fluoroethyl azide, CuI, NEt_3_, *N-*iodosuccinimide, MeCN, 3 h, r.t.; (**b**) 4 M HCl, MeCN, reflux, 1 h.; (**c**) [^123/125^I]NaI, bathophenanthroline/CuCl_2,_ NEt_3_, 2-fluoroethyl azide, MeCN, 60 min, r.t.; (**d**) [^125^I]NaI, bathophenanthroline/CuCl_2_, 2-fluoroethyl azide, MeCN, 60 min, r.t.

The non-radioactive reference compound, (S)-5-((2-((2,4-difluorophenoxy)methyl)pyrrolidin-1-yl)sulfonyl)-1-((1-(2-**F**luoroethyl)-5-(**I**odo)-1H-1,2,3-**T**riazol-4-yl)methyl)**I**ndoline-2,3-dione (referred to as FITI) was prepared in two steps. Treatment of the acetal protected isatin **1**^30^ with 2-fluoroethyl azide^35^ in the presence of copper(I) iodide, triethylamine and *N*-iodosuccinimide, according to a previously reported method for formation of iodotriazoles (Fig. 2)^36^, gave the resulting 5-iodo-triazole **2**. Subsequent deprotection with aqueous HCl afforded the target compound FITI in 64% overall yield. The alkyne **3** was obtained in 97% yield by alkylation of isatin with propargyl bromide. Subsequent reaction of **3** with 2-fluoroethyl azide and *N*-iodosuccinimide afforded the 5-iodo-1,2,3-triazole **4**, which served as a negative control for biological assays.

**Figure 3:**
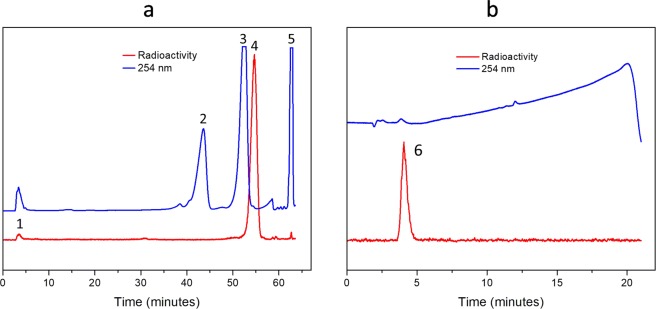
HPLC Chromatograms. (**a**) Preparative HPLC profile of the reaction mixture following labelling of [^125^I]FITI, showing residual [^125^I]iodide (1), ICMT-11 (2), alkyne **5** (3), [^125^I]FITI (4), and BPhen complex (5). (**b**) Analytical HPLC profile of [^125^I]FITI after purification and formulation in saline/10% EtOH (v/v) (6) [NB: The HPLC systems used for (a) and (b) are different].

Attempted labelling of FITI by reaction of the isatin **5** with [^125^I]iodide in the presence of 2-fluoroethyl azide, using previously reported reaction conditions (60 °C, CuCl_2_, Et_3_N)^36^, was unsuccessful and led to decomposition of the precursor **5**. Eventually, we were able to resolve this problem by employing bathophenanthroline (BPhen) as a copper(I) chelator. We have previously shown that inclusion of bathophenanthrolinedisulfonate (BPDS) accelerates the copper(I) catalysed cyclisation reaction of 2-fluoroethyl azide with alkynes, and that it also can perturb side reactions by disrupting unfavourable interactions between copper(I) and other functional groups^29,37^. Gratifyingly, this strategy also proved highly effective for the three-component click reaction, and in this case addition of BPhen to the labelling reaction allowed formation of [^125^I]FITI from isatin **5** at room temperature within 1 h in >95% analytical yield as determined by HPLC. The isolated radiochemical yield after formulation was 55 ± 12% (*n* = 8) with a radiochemical purity of >99%. The molar activity was >1.1 GBq/µmol. Figure 3 shows typical HPLC chromatograms of the crude labelling mixture, and [^125^I]FITI after purification. The iodine-123 labelled analog [^123^I]FITI was obtained in 51% (*n = *2) decay corrected radiochemical yield after formulation with a radiochemical purity >99% and molar activity >4.3 GBq/µmol (based on the limit of UV signal quantification). The total time of radiosynthesis including formulation was 3 hours.

### *In vitro* binding affinity

**Figure 4:**
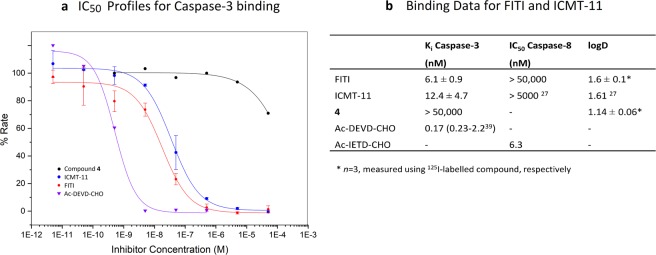
*In vitro* data. (**a**) IC_50_ profiles for caspase-3 binding of FITI, ICMT-11, compound **4**, and Ac-DEVD-CHO. Data for compound **4** and Ac-DEVD-CHO are averages of three experiments from one assay plate. Data for FITI and ICMT-11 are averages of three assay plates from three experiments each with standard deviations shown. (**b**) Table showing caspase-3 and caspase-8^39^ binding data for FITI, ICMT-11 and compound **4**.

To determine the affinity of FITI for caspase-3 we used a commercially available enzyme kit, as previously described^27^. ICMT-11 and the peptide inhibitor Ac-DVD-CHO^38^ were included as positive controls, and the 5-iodo-1,2,3-triazole **4** used as a negative control for the assay. Unexpectedly, we were initially unable to obtain reproducible results with ICMT-11, which gave IC_50_ readouts in the micromolar range. Systematic investigation of the assay conditions revealed that dithiothreitol (DTT), an additive present in the buffer supplied with the enzyme kit, rapidly reacted with ICMT-11 (incubation of ICMT-11 with the enzyme assay buffer led to its complete conversion to unknown products within 10 min at 40 °C as determined by HPLC). Consistent with this observation, the use of a DTT-free buffer (prepared according to Kopka *et al*.^22^ but without 2-mercaptoethanol) gave robust readouts from the assay (Fig. 4) with Ki values of 6.1 ± 0.9 nM and 12.4 ± 4.7 nM for FITI and ICMT-11, respectively. Caspase-8 was also included in the assay to evaluate the isoform selectivity, and for this enzyme no appreciable affinity was observed for FITI (IC_50_ > 50 µM vs 6.3 nM for the peptide inhibitor Ac-IETD-CHO). The results from the enzyme assay are consistent with our hypothesis that the 5-iodo-1,2,3-triazole moiety can interact with both polar and lipophilic domains of the caspase-3 S1 binding pocket (Fig. 1). This is intriguing as the S1 pocket previously have been targeted with benzyl as well as a range of aryl groups, and our finding therefore raises the prospect of using trifunctionalized triazoles as highly flexible bioisosteres for one of the most commonly used motifs in medicinal chemistry.

### Biodistribution studies

**Figure 5:**
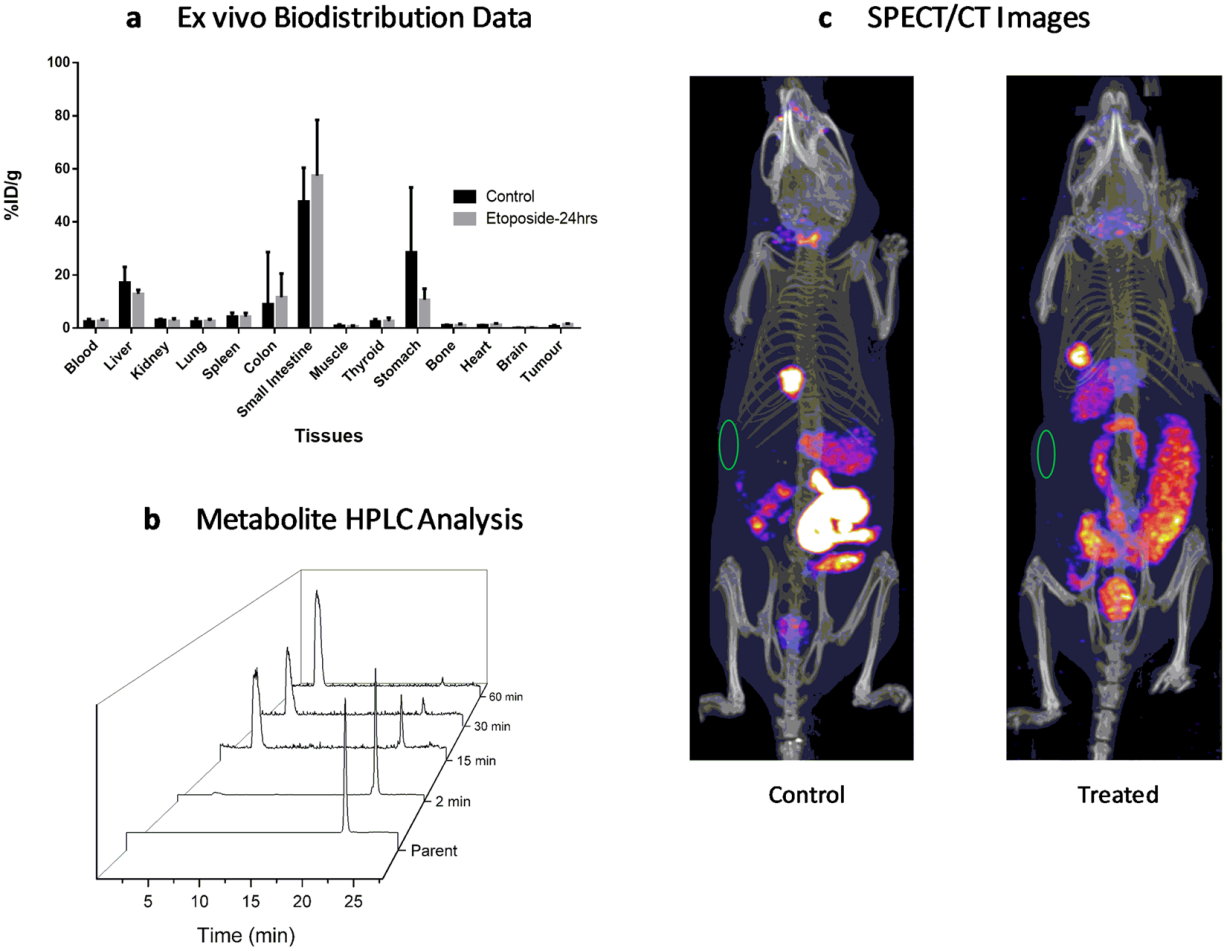
Biology data. (**a**) *Ex vivo* biodistribution data for selected tissues in control and etoposide-treated mice 60 min after [^125^I]FITI injection into SW1222 tumour-bearing mice. (**b**) HPLC profiles of mice plasma metabolite samples showing degradation of [^125^I]FITI (t_R_ = 22.3 min). (**c**) SPECT/CT images of SW1222 tumour mice using [^123^I]FITI. Control and etoposide treated mouse after 24 h, the green circle indicates the location of the xenograft. Both animals have been injected with 5 MBq of [^123^I]FITI.

In exploratory *in vitro* studies we identified SW1222 as a promising cell line for imaging of drug induced apoptosis with etoposide. Biodistribution of [^125^I]FITI was therefore determined in SW1222 tumour-bearing animals 60 min after *i.v*. tail vein injection in either etoposide-treated or control animals (Fig. 5). In control animals, the tracer showed high uptake in the small intestine (48 ± 13%ID/g), stomach (28 ± 25%ID/g) and liver (17 ± 6%ID/g), and low uptake in the tumour (0.7 ± 0.4%ID/g). The distribution profile suggest that clearance of [^125^I]FITI was dominated by the hepatobiliary route. This is mirrored by the pre-clinical and first-in-man results with [^18^F]ICMT-11^28,31^. These data also indicate that de-iodination might not be the primary cause of increased intestinal uptake with [^125^I]FITI, and may instead be due to caspase-3 expression in apoptotic intestinal cells.

Mice treated with a single dose of etoposide (50 mg/kg, 24 hrs, i.p.) demonstrated significantly higher tumour levels of [^125^I]FITI compared to control animals (1.5 ± 0.2 vs 0.7 ± 0.4%ID/g) (p < 0.001). However, the overall uptake in the tumour was too low to allow *in vivo* imaging of tumour apoptosis with SPECT. The biodistribution study did not reveal any other substantial changes in tracer uptake following etoposide treatment, although *in vivo* SPECT imaging showed marked changes in the abdominal distribution of FITI. It is unclear if this reflects drug induced apoptosis, or if the observed changes were due to swelling or increased local blood flow. Analysis of the mouse plasma following *i.v*. injection of [^125^I]FITI indicated rapid metabolism with less than 50% intact parent tracer present 15 min post-injection (Fig. 5). A single polar radioactive metabolite was detected consistent with deiodination *in vivo* to give [^125^I]iodide. However, it should be noted that a similar polar metabolite has been observed for the non-iodinated analog [^18^F]ICM-11, and the low uptake in the thyroid (2.4 ± 1%ID/g at 60 min post-injection) and high liver uptake point to another metabolic route. In any case, the rapid metabolism makes FITI poorly suited as a tracer for *in vivo* imaging of apoptosis.

## Conclusions

We have prepared the 5-iodo-1,2,3-triazole analog of ICMT-11 as a putative dual SPECT and PET tracer for imaging of drug-induced apoptosis. The compound showed low nanomolar affinity for caspase-3, and increased potency as compared to ICMT-11. The results are consistent with our hypothesis that the 5-iodo-1,2,3-triazole moiety interacts with both polar and lipophilic domains of the caspase-3 S1 binding pocket. The radioiodinated tracer was obtained in 55% radiochemical yield using, for the first time, chelator-accelerated copper catalysed three-component click chemistry for preparation of 5-iodo-1,2,3-triazoles. Biological characterisation of the tracer showed increased tumour uptake in etoposide treated mice, as compared with the non-treated control group. However, low tumour uptake and rapid metabolism precludes the use of the tracer for imaging of tumour apoptosis *in vivo*.

## Methods

All methods and data for synthetic chemistry, radiochemistry and biology are available in supporting information.

### Chemistry

#### (*S*)-5-((2-((2,4-difluorophenoxy)methyl)pyrrolidin-1-yl)sulfonyl)-1-((1-(2-fluoroethyl)-5-iodo-1*H*-1,2,3-triazol-4-yl)methyl)indoline-2,3-dione (FITI)

Alkyne **1** (25 mg, 54 µmol)^27^ was dissolved in acetonitrile (500 µL). To the resulting solution were added copper(I) iodide (9 mg, 47 µmol), triethylamine (7 µL, 50 µmol), 2-fluoroethyl azide as a 0.5 M solution in THF (100 µL, 50 µmol), and *N*-iodosuccinimide (12 mg, 53µmol). The mixture was stirred at room temperature for 3 hours. After evaporating the solvent using a stream of nitrogen, the residue was dissolved in ethyl acetate, filtered through Celite^®^, and purified using column chromatography (SiO_2_, 50% ethyl acetate in *n*-hexane). The resulting pure isolated fraction of intermediate **2** was re-dissolved in acetonitrile (2 mL) and a 4 M aqueous solution of hydrochloric acid (1 mL, 4 mmol) was added. The resulting mixture was heated to reflux for 1 h. After cooling to room temperature, the reaction mixture was diluted with water (25 mL) and the product was extracted with ethyl acetate (3 × 25 mL). The combined organic layers were dried over magnesium sulfate and the solvent was evaporated under reduce pressure to afford the tittle compound as a yellow crystals (21 mg, 62%); ^**1**^**H NMR (600 MHz, CDCl**_**3**_**)** δ (ppm): 8.05 (dd, ^3^*J* = 8.3 Hz, ^4^*J* = 1.9 Hz, 1 H, Ph-*H*6), 8.02 (d, ^4^*J* = 1.9 Hz, 1 H, Ph-*H*4), 7.49 (d, ^3^*J* = 8.3 Hz, 1 H, Ph-*H*7), 6.93 (td, *J* = 9.2 Hz, 5.2 Hz, 1 H, Ar*H*), 6.84–6.77 (m, 2 H, Ar*H*), 5.06 (s, 2 H, -NC*H*_2_Triazole), 4.86 (dt, ^2^*J*_H-F_ = 46 Hz, ^3^*J* = 5.1 Hz, 2 H, Triazole-CH_2_C*H*_2_F), 4.69 (dt, ^3^*J*_H-F_ = 24 Hz, ^3^*J* = 5.1 Hz, 2 H, Triazole-C*H*_2_CH_2_F), 4.20 (dd, *J* = 9.2 Hz, 3.1 Hz, 1 H, *H*14_α_), 3.99–3.95 (m, 1 H, *H*14_β_), 3.94–3.92 (m, 1 H, *H*13), 3.50–3.52 (m, 1 H, Pyrro-*H*2_eq_), 3.13–3.17 (m, 1 H, Pyrro-*H*2_ax_), 2.08-1.98 (m, 2 H, Pyrro-*H*3,4_ax_), 1.82–1.73 (m, 2 H, Pyrro-*H*3,4_eq_); ^**13**^**C NMR (150 MHz, CDCl**_**3**_**)** δ (ppm): 181.5 (C), 157.2 (C), 156.7 (dd, *J*_C-F_ = 242 Hz, 10 Hz, C), 153.1 (C), 152.3 (dd, *J*_C-F_ = 249 Hz, 11 Hz, C), 145.6 (C), 143.1 (dd, *J*_C-F_ = 10 Hz, 3 Hz, C), 137.6 (CH), 133.9 (C), 124.4 (CH), 117.5 (C), 115.5 (dd, *J*_C-F_ = 9 Hz, 2 Hz, CH), 112.5 (CH), 110.6 (dd, *J*_C-F_ = 22 Hz, 5 Hz, CH), 105.0 (dd, *J*_C-F_ = 27 Hz, 22 Hz, CH), 81.2 (C), 81.1 (d, *J*_C-F_ = 172 Hz, CH_2_), 71.6 (CH_2_), 58.7 (CH), 51.0 (d, *J*_C-F_ = 22 Hz, CH_2_), 49.6 (CH_2_), 36.2 (CH_2_), 29.0 (CH_2_), 24.2 (CH_2_); **m.p.:** 163–165 °C; **HRMS (ESI):** [M + H]^+^ Calcd. m/z 676.0339, found m/z 676.0359.

### Radiochemistry

#### Radiolabelling of [^123, 125^I]FITI

To a suspension of copper(II) chloride (134 µg, 1.0 µmol) in anhydrous acetonitrile (20 µL) and triethylamine (151 µg, 1.5 µmol) was added a solution of bathophenanthroline (BPhen) (33 µg, 0.1 µmol) in anhydrous acetonitrile (20 µL). A solution of the isatin alkyne **5** (460 µg, 1.0 µmol) in anhydrous acetonitrile (20 µL) was subsequently added and the mixture was left for 5 min at room temperature. *NB: The isatin alkyne/CuCl*_2_*/bathophenanthroline/triethylamine complex in acetonitrile must be freshly prepared and used immediately. Otherwise, the reaction will not work, or give low RCY*. The resulting solution was added to aqueous [^125^I]NaI (1–37 MBq, 6.0 µL) in a polypropylene (PP) centrifuge tube (1.5 mL) immediately followed by a stock solution of 2-fluoroethyl azide in anhydrous acetonitrile (40 mM, 25 µL, 1 µmol). The tube was kept at room temperature with occasional agitation for 60 min. The reaction mixture was diluted with a solution of acetonitrile and water (0.5 mL, 3:2 v/v). The resulting solution was purified by HPLC on a ZORBAX® column (300SB-C18, 5 µm, 9.4 × 250 mm, Agilent) using water and methanol, each containing 0.1% TFA (gradient elution with a flow rate of 3 mL/min, from 40% to 55% methanol content over 50 min, then from 55% to 90% methanol content over 5 min; Fig. 3, R_t_ ≈ 54 min). The collected fraction was diluted with water (5 mL), and loaded on a conditioned Sep-Pak tC18 Plus Light Cartridge. After washing with water (5 mL) and drying with air (15 mL), the cartridge was eluted with EtOH (0.5 mL). The product was collected in fractions (0.1 mL). The fractions containing >90% of radioactivity (typically fractions 2 and 3) were combined and diluted with saline to give an EtOH content of < 10% (v/v). The final sample was obtained with a radiochemical yield of 55 ± 12% (*n = *8) and a radiochemical purity of >99%. The iodine-123 labelled analog was obtained using an otherwise identical procedure with the exception that aqueous [^123^I]NaI (6 µL, 48–132 MBq) was used. The target compound [^123^I]FITI was obtained in a decay-corrected radiochemical yield of 51% (*n* = 2), and with a radiochemical purity >99%.

### Biology

#### Caspase enzyme inhibition assay

Recombinant human caspase-3 and caspase-8 enzymes, substrates Ac-DEVD-AMC and Ac-IETD-AMC, and inhibitors Ac-DEVD-CHO and Ac-IETD-CHO were purchased from Enzo Life Sciences AG. The assay buffer was consisted of HEPES (pH 7.4, 20 mM), sucrose (10%), NaCl (100 mM), CHAPS (0.1%), and EDTA (2 mM). A Varioskan-LUX (ThermoFisher UK) fluorescence plate reader was used to read 96-well plates in triplicate at 37 °C incubation temperature (excitation wavelength = 355 nm, emission wavelength = 460 nm). The inhibitor test compounds were serially diluted in DMSO (1 mM, 0.1 mM, 10 µM, 1 µM, 0.1 µM, 10 nM, 1 nM, and 0.1 pM). The caspase enzyme stock solutions were made up to give 1 U per well. The substrates were prepared in assay buffer solution (40 µM). Inhibitor solution (5 µL) was added to wells containing assay buffer (20 µL) followed by substrate solution (50 µL). After an incubation period at 37 °C for 10 min, the enzyme solution was added (25 µL). The wells of the blank experiments contained assay buffer (50 µL) and substrate solution (50 µL). The control wells used assay buffer (20 µL), enzyme solution (25 µL), substrate (50 µL), and DMSO (5 µL). Fluorescence signals were measured once per minute for one hour. The IC50 values were computed based on the linear ranges of the dynamic signal profiles using non-linear correlation software (OriginPro 2015). The Ki values were calculated from an average of three IC50 measurements using the Cheng-Prusoff equation and a Km for Ac-DEVD-AMC of 9.7 µM.

#### Biodistribution studies in tumour-bearing mice

Biodistribution studies in tumour-bearing mice were carried out using female NOD scid-gamma (NSG) mice (6–8 weeks old, 20–25 g) bred in the UCL animal facility. For all studies mice were acclimatized for a week prior to initiation of studies. Mice were housed under sterile conditions in individually ventilated cages, fed with standard chow diet and water ad libitum and maintained on an automatic 12 h light cycle at 22–24 °C. All animal experiments were performed in accordance with the UK Home Office Animals Scientific Procedures Act 1986 and United Kingdom Co-ordinating Committee on Cancer Research Guidelines for the Welfare and Use of Animals in Cancer Research, and were approved by the University College London Animal Ethics Committee. Subcutaneous tumours were established in female NSG mice using human colorectal adenocarcinoma cell line SW1222 cells. Briefly, 5 × 106 cells were injected into the right flank of mice and allowed to grow to a volume of 0.3–0.5 cm^3^. Tumour volumes were calliper-measured and calculated using the formula: volume = 4π/3 (1/2 length × 1/2 width × 1/2 height). Tumours were sized-matched and animals were either untreated or treated with a single dose of clinically formulated etoposide 24 h before radiotracer injection (Eposin, 50 mg/kg, i.p.).

#### Plasma metabolite analysis

Blood (0.7–1.0 mL) was removed from BALB/c mice shortly after the animals were euthanized and was spun down in a micro-centrifuge at 13,000 rpm for 3 min. The supernatant was decanted and mixed with ice-cold MeCN (1.0 mL) followed by vortexing (1 min) and centrifugation (13,000 rpm, 3 min). The supernatant was diluted with water (1.0 mL) and analyzed by HPLC on a ZORBAX® column (300SB-C18, 5 µm, 9.4 × 250 mm, Agilent) using water and methanol, each containing 0.1% TFA (gradient elution with a flow rate of 3 mL/min, hold 1 min at 10% methanol content and then from 10% to 90% methanol content over 20 min).

#### SPECT-CT imaging of [^123^I]FITI in SW1222 tumour-bearing mice

The mice were anesthetised by inhalation of 2% isoflurane in oxygen, injected intravenously via the tail vein with 5 MBq of [^123^I]FITI, and imaged with a NanoSPECT/CT small animal imager (Mediso Imaging Systems, Budapest, Hungary) for 2 h using a multi-pinhole (nine pinholes, aperture 1.0 mm collimator and a transaxial FOV of 62 mm). Single photon emission computed tomography images were reconstructed using Hi SPECT software and a dedicated ordered subset-expectation maximisation algorithm (Scivis, Goettingen, Germany).

### Statistical analysis

Data are expressed as mean ± SD and comparison between 2 datasets was determined using the Student t test (Prism v6.0 software, GraphPad). Results were considered as statistically significant at a p-value of ≤ 0.05.

## Supplementary information

Supplementary information

## Data Availability

All data generated or analysed during this study are included in this published article and the Supplementary Information.
