# Supplementary material for: One-Pot Radiosynthesis and Biological Evaluation of a Caspase-3 Selective 5-[123,125I]iodo-1,2,3-triazole derived Isatin SPECT Tracer
Source: Sci Rep. 2019 Dec 17;9:19299. doi: 10.1038/s41598-019-55992-0 (PMC6917698; doi:10.1038/s41598-019-55992-0)
Supplement: Supplementary file 1 — Supplementary information [file 41598_2019_55992_MOESM1_ESM.pdf]

Supplementary Information

One-Pot Radiosynthesis and Biological Evaluation of a Caspase-3 Selective 5-[^{123,125}I]iodo-1,2,3-triazole derived Isatin SPECT Tracer

Matthias Glaser,[1,2] Vineeth Rajkumar,[3] Seckou Diocou,[3] Thibault Gendron,[1,2] Ran Yan,[4] Pak Kwan Brian Sin,[1] Kerstin Sander,[1,2] Laurence Carroll,[5] R. Barbara Pedley,[3] Eric O. Aboagye,[5] Timothy H. Witney,[4,6] Erik Årstad[1,2]

[1] Centre for Radiopharmaceutical Chemistry, University College London, 5 Gower Place, London WC1E 6BS, United Kingdom.
[2] Department of Chemistry, University College London, 20 Gordon Street, London, WC1H 0AJ, United Kingdom
[3] UCL, Cancer Institute, 72 Huntley Street, London, WC1E 6DD, UK
[4] King's College London, School of Biomedical Engineering and Imaging Sciences, St. Thomas' Hospital, SE1 7EH, London, United Kingdom
[5] Imperial College London, Science, Technology & Medicine, Department of Medicine, Hammersmith Hospital, DuCane Road, London W12 0NN, United Kingdom
[6] Centre for Advanced Biomedical Imaging, Division of Medicine, University College London, London, United Kingdom

Table of Content

1. Organic Chemistry S2
   1.1. General considerations S2
   1.2. Synthesis of the non-radioactive reference FITI S3
   1.3. Synthesis of compounds **3** and **4** S5
2. Radiolabeling experiments S8
   2.1. Definitions and general considerations S8
   2.2. Cartridge conditioning S8
   2.3. Radiolabeling of [$^{123/125}$I]FITI S8
   2.4. Radiolabeling of [^{125}I]**4** S9
   2.5. Log D Measurement S10
3. Biology S10
   3.1. Caspase enzyme inhibition assay S10
   3.2. Plasma metabolite analysis S11
   3.3. Biodistribution studies in mice S11
   3.4. Tumour Models S11
   3.5. Biodistribution of [^{125}I]FITI in SW1222 tumour-bearing mice S11
   3.6. SPECT-CT imaging of [^{123}I]FITI in SW1222 tumour-bearing mice S12
   3.7. Statistical analysis S12
References S13

1. Organic Chemistry

1.1. General considerations

Commercially available starting materials were purchased from Sigma-Aldrich, Alfa Aesar, and Acros Organics and were used without further purification. Solvents were obtained from Sigma-Aldrich; unless stated otherwise, reagent grade solvents were used for reactions and column chromatography and analytical grade solvents were used for recrystallizations. Reaction progress was monitored by thin layer chromatography (TLC) on aluminium sheets coated with silica gel 60 F_{254} (Merck Millipore) and detection was carried out using UV light (325 nm and 254 nm) and/or chemical solutions. Crude reaction mixtures were purified either by recrystallization or by manual flash column chromatography on silica gel 60 (230-400 mesh, 0.040-0.063 mm; Merck Millipore).

^{1}H and ^{13}C Nuclear Magnetic Resonance (NMR) spectra were recorded on a Bruker Avance 300, 400, or 600 at room temperature. ^{13}C NMR experiments were proton decoupled. All spectra were internally referenced to the respective deuterated solvents. Chemical shifts are reported in ppm and coupling constants (*J*) are given in Hertz (Hz). Multiplicity is described with (s): singlet, (d): doublet, (t): triplet and (q): quadruplet. NMR assignment was performed with the aid of multidimensional and long-range experiments. Carbon multiplicities were assigned by Distortionless Enhancement by Polarization Transfer (DEPT) experiments and are reported as follows, (C): quaternary carbon, (CH_2): secondary carbon, (CH_3): methyl group. Assignment on phenyl rings and pyrrolidine rings are reported with the abbreviations Ph- and Pyrro-, respectively.

High resolution mass data were recorded on a Thermo Finnigan MAT900xp (CI, EI), Waters LCT Premier spectrometers using positive electrospray ionization (ESI). Melting points were taken on a Gallenkamp heating block and are uncorrected.

1.2. Synthesis of the non-radioactive reference FITI

(*S*)-5-((2-((2,4-difluorophenoxy)methyl)pyrrolidin-1-yl)sulfonyl)-1-((1-(2-fluoroethyl)-5-iodo-1*H*-1,2,3-triazol-4-yl)methyl)indoline-2,3-dione (FITI)

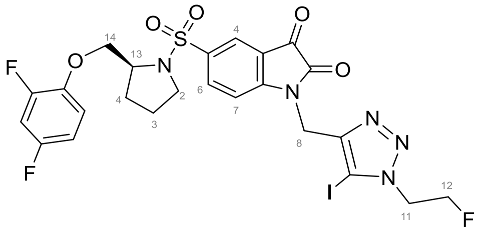

Appearance: yellow crystals
Chemical Formula: $C_{24}H_{21}F_3IN_5O_5S$
Molecular Weight: 675.42
Yield: 62%

Alkyne **1** (25 mg, 54 µmol)[1] was dissolved in acetonitrile (500 µL). To the resulting solution were added copper(I) iodide (9 mg, 47 µmol), triethylamine (7 µL, 50 µmol), 2-fluoroethyl azide as a 0.5M solution in THF (100 µL, 50 µmol), and *N*-iodosuccinimide (12 mg, 53µmol). The mixture was stirred at room temperature for 3 hours. After evaporating the solvent using a stream of nitrogen, the residue was dissolved in ethyl acetate, filtered through Celite®, and purified using column chromatography (SiO_2, 50% ethyl acetate in *n*-hexane). The resulting pure isolated fraction of intermediate **2** was re-dissolved in acetonitrile (2 mL) and a 4M aqueous solution of hydrochloric acid (1 mL, 4 mmol) was added. The resulting mixture was heated to reflux for 1 h. After cooling to room temperature, the reaction mixture was diluted with water (25 mL) and the product was extracted with ethyl acetate (3×25 mL). The combined organic layers were dried over magnesium sulfate and the solvent was evaporated under reduce pressure to afford the tittle compound as yellow crystals (21 mg, 62%).

^{1}H NMR (600 MHz, $CDCl_3$) δ (ppm): 8.05 (dd, ^3J = 8.3 Hz, ^4J = 1.9 Hz, 1H, Ph-*H*6), 8.02 (d, ^4J = 1.9 Hz, 1H, Ph-*H*4), 7.49 (d, ^3J = 8.3 Hz, 1H, Ph-*H*7), 6.93 (td, *J* = 9.2 Hz, 5.2 Hz, 1H, Ar*H*), 6.84-6.77 (m, 2H, Ar*H*), 5.06 (s, 2H, -NCH_2Triazole), 4.86 (dt, $^2J_{H\text{-}F}$ = 46 Hz, ^3J = 5.1 Hz, 2H, Triazole-CH_2CH_2F), 4.69 (dt, $^3J_{H\text{-}F}$ = 24 Hz, ^3J = 5.1 Hz, 2H, Triazole-CH_2CH_2F), 4.20 (dd, *J* = 9.2 Hz, 3.1 Hz, 1H, $H14_{\alpha}$), 3.99-3.95 (m, 1H, $H14_{\beta}$), 3.94-3.92 (m, 1H, *H*13), 3.50-3.52 (m, 1H, Pyrro-$H2_{eq}$), 3.13-3.17 (m, 1H, Pyrro-$H2_{ax}$), 2.08-1.98 (m, 2H, Pyrro-$H3,4_{ax}$), 1.82-1.73 (m, 2H, Pyrro-$H3,4_{eq}$)

^{13}C NMR (150 MHz, $CDCl_3$) δ (ppm): 181.5 (C), 157.2 (C), 156.7 (dd, $J_{C\text{-}F}$ = 242 Hz, 10 Hz, C), 153.1 (C), 152.3 (dd, $J_{C\text{-}F}$ = 249 Hz, 11 Hz, C), 145.6 (C), 143.1 (dd, $J_{C\text{-}F}$ = 10 Hz, 3 Hz, C), 137.6 (CH), 133.9 (C), 124.4 (CH), 117.5 (C), 115.5 (dd, $J_{C\text{-}F}$ = 9 Hz, 2 Hz, CH), 112.5 (CH), 110.6 (dd, $J_{C\text{-}F}$ = 22 Hz, 5 Hz, CH), 105.0 (dd, $J_{C\text{-}F}$ = 27 Hz, 22 Hz, CH), 81.2 (C), 81.1 (d, $J_{C\text{-}F}$ = 172 Hz, CH_2), 71.6 (CH_2), 58.7 (CH), 51.0 (d, $J_{C\text{-}F}$ = 22 Hz, CH_2), 49.6 (CH_2), 36.2 (CH_2), 29.0 (CH_2), 24.2 (CH_2)

m.p.: 163-165 °C

HRMS (ESI): $[M+H]^+$ Calcd. m/z 676.0339, found m/z 676.0359

(*S*)-5-((2-((2,4-difluorophenoxy)methyl)pyrrolidin-1-yl)sulfonyl)-1-((1-(2-fluoroethyl)-5-iodo-1*H*-1,2,3-triazol-4-yl)methyl)indoline-2,3-dione (**FITI**) (^{1}H NMR, $CDCl_3$, 600 MHz)

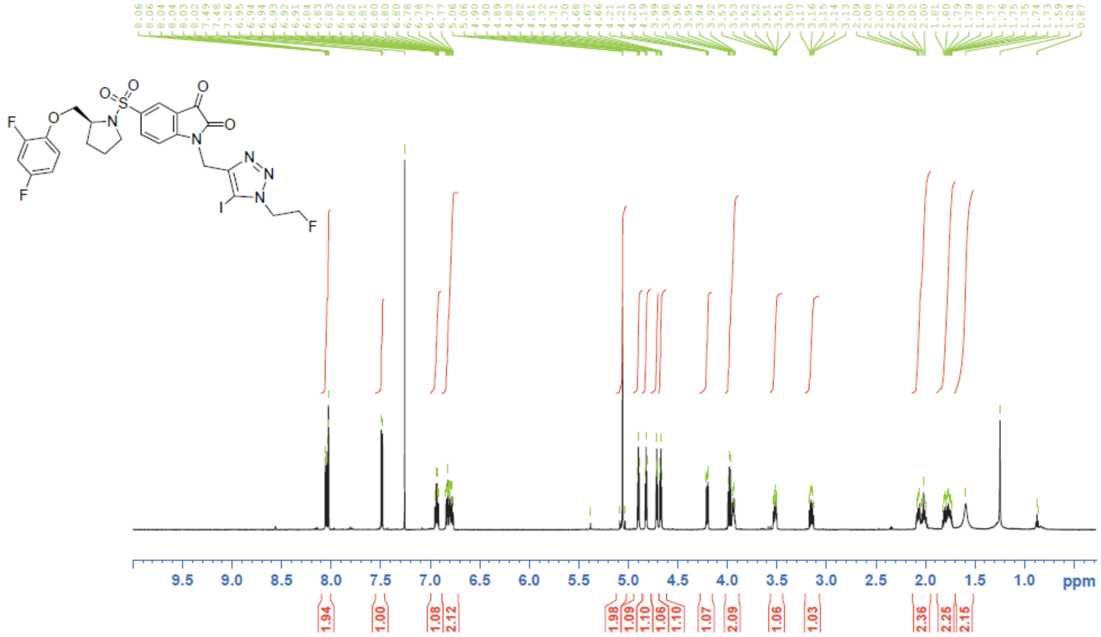

(*S*)-5-((2-((2,4-difluorophenoxy)methyl)pyrrolidin-1-yl)sulfonyl)-1-((1-(2-fluoroethyl)-5-iodo-1*H*-1,2,3-triazol-4-yl)methyl)indoline-2,3-dione (**FITI**) (^{13}C NMR, $CDCl_3$, 150 MHz)

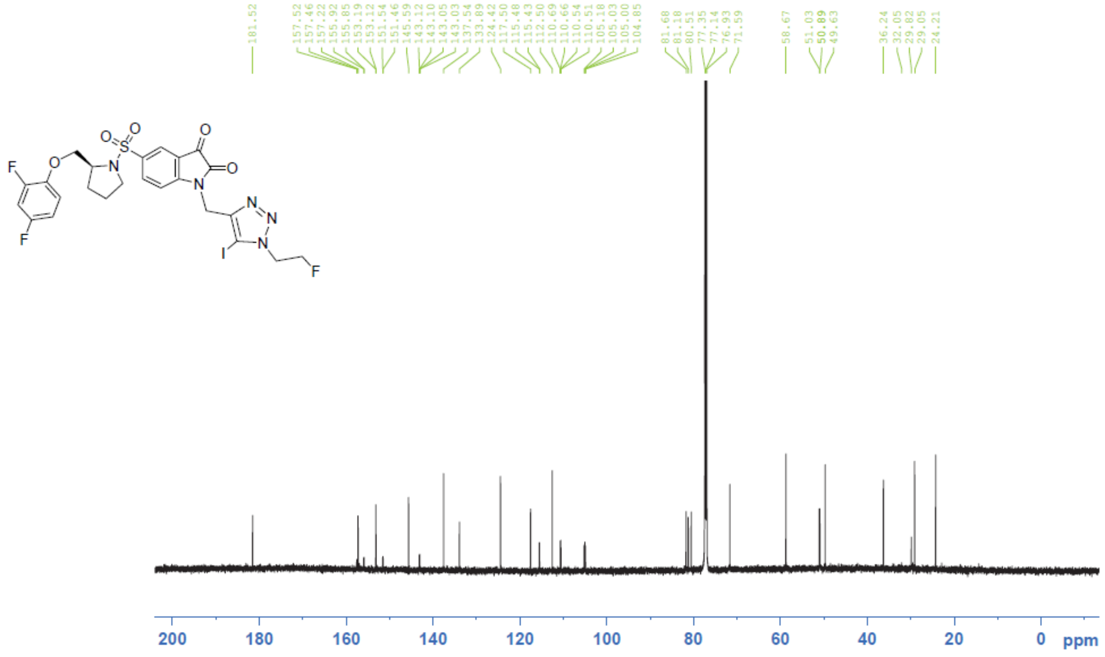

1.3. Synthesis of compounds 3 and 4

1-(Prop-2-yn-1-yl)indoline-2,3-dione (3)

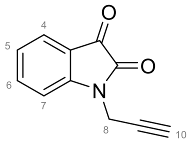

Appearance: Orange solid
Chemical Formula: $C_{11}H_7NO_2$
Molecular Weight: 185.18
Yield: 97%

Isatin (400 mg, 2.72 mmol) and cesium carbonate (975 mg, 2.99 mmol) were dissolved in dimethylformamide (10 mL) and the reaction mixture was stirred at ambient temperature for 2 h. To this was added a solution of propargyl bromide in toluene (80%w/w, 304 µL, 3.26 mmol) and the reaction was stirred further for 1 h. Solvents and excess propargyl bromide were removed *in vacuo* by co-evaporating with toluene. The crude residue was purified by column chromatography (SiO_2, 10%→40% EtOAc in petrol) to afford the titled product as an orange solid (490 mg, 97%).

^{1}H NMR (600 MHz, $CDCl_3$) δ (ppm): 7.67-7.63 (m, 2H, Ar*H*), 7.18 (t, ^3J = 7.5 Hz, 1H, Ar*H*), 7.14 (d, ^3J = 8.2 Hz, 1H, Ar*H*), 4.54 (d, ^4J = 2.5 Hz, 2H, -CH_2-), 2.31 (t, ^4J = 2.5 Hz, 1H, CC*H* alkyne)
^{13}C NMR (150 MHz, $CDCl_3$) δ (ppm): 182.7 (C), 157.3 (C), 149.7 (C), 138.6 (CH), 125.6 (CH), 124.4 (CH), 117.8 (C), 111.2 (CH), 75.8 (C), 73.5 (CH), 29.6 (CH_2)
m.p.: 156-160 °C
HRMS (ESI): $[M]^+$ Calcd. m/z 185.0471, found m/z 185.0472

1-(prop-2-yn-1-yl)indoline-2,3-dione (**3**) (^{1}H NMR, $CDCl_3$, 600 MHz)

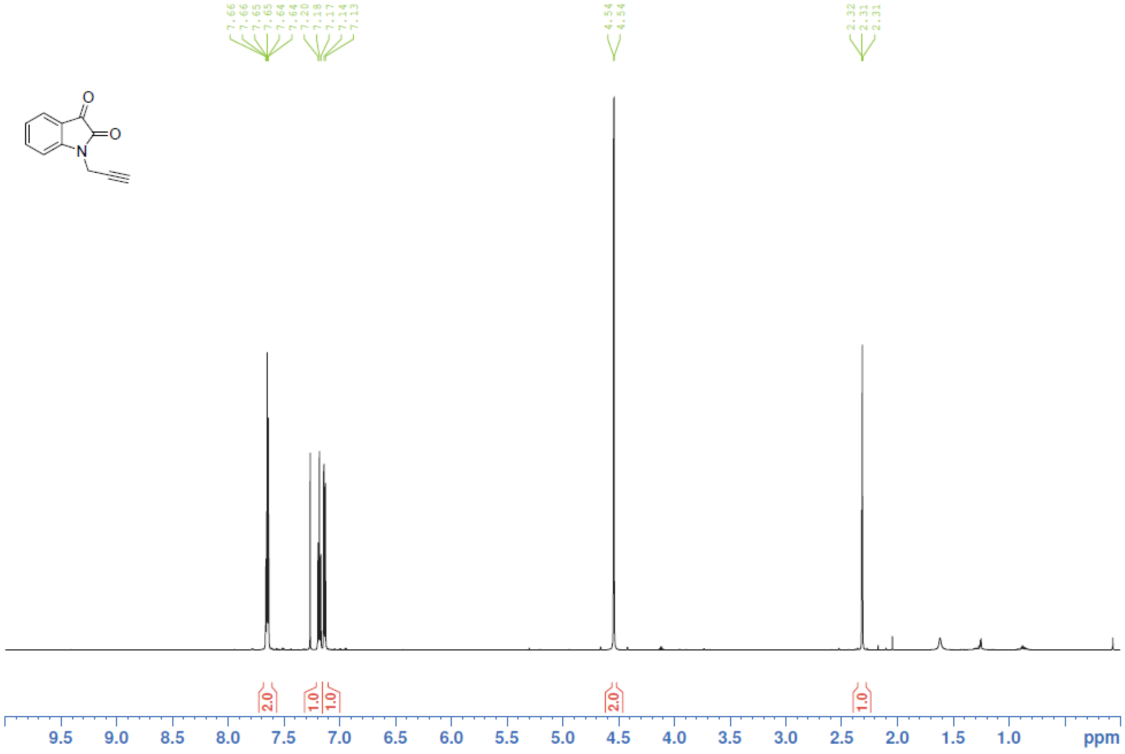

1-(prop-2-yn-1-yl)indoline-2,3-dione (**3**) (^{13}C NMR, $CDCl_3$, 150 MHz)

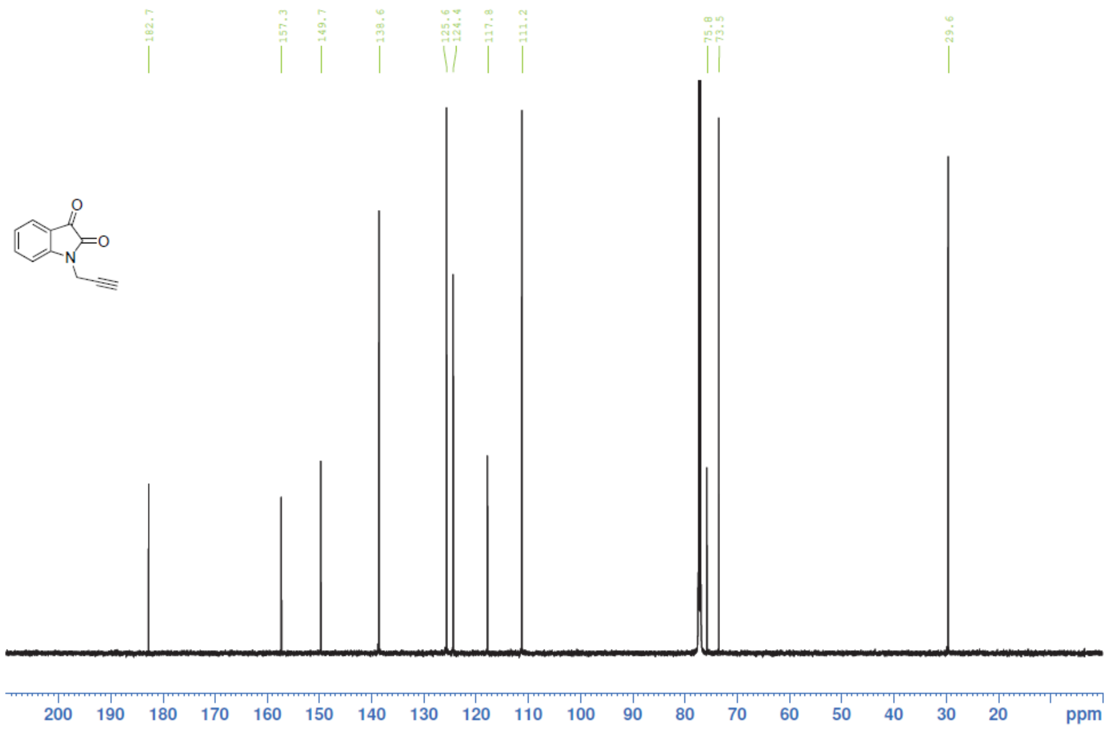

1-((1-(2-Fluoroethyl)-5-iodo-1*H*-1,2,3-triazol-4-yl)methyl)indoline-2,3-dione (4)

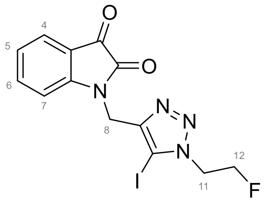

Appearance: Orange crystals
Chemical Formula: $C_{13}H_{10}FIN_4O_2$
Molecular Weight: 400.15
Yield: 27%

The alkyne **3** (20 mg, 108 µmol) was dissolved in anhydrous DMF (100 µL) and mixed with a suspension of copper(I) iodide (21 mg, 108 µmol) and triethylamine (15 µL, 108 µmol) in anhydrous DMF (100 µL). A solution of 2-fluoroethylazide (0.4 M in DMF, 270 µL, 108 µmol) was added to the reaction mixture with stirring, followed by addition of a solution of *N*-iodosuccinimide (27 mg, 119 µmol) in anhydrous DMF (100 µL). After stirring for 2.5 hours at room temperature, the reaction mixture was quenched with water (10 mL), and extracted with ethyl acetate (5 mL). The extract was washed with brine (2 mL) and dried over magnesium sulfate. The product was purified using re-crystallization from ethyl acetate to form orange needles (11.7 mg, 27%)

^{1}H NMR (600 MHz, DMSO-*d*$_6$) δ (ppm): 7.66 (td, ^3J = 7.7 Hz, ^4J = 1.5 Hz, 1H, Ph-*H*6), 7.59 (dd, ^3J = 7.7 Hz, ^4J = 0.8 Hz, 1H, Ph-*H*4), 7.16 (t, ^3J = 7.7 Hz, 1H, Ph-*H*5), 7.14 (dd, ^3J = 7.7 Hz, ^4J = 0.8 Hz, 1H, Ph-*H*7), 4.95 (s, 2H, -NCH_2Triazole), 4.81 (dt, $^2J_{H-F}$ = 47 Hz, ^3J = 4.7 Hz, 2H, Triazole-CH_2CH_2F), 4.69 (dt, $^3J_{H-F}$ = 26 Hz, ^3J = 4.7 Hz, 2H, Triazole-CH_2CH_2F)
^{13}C NMR (150 MHz, DMSO-*d*$_6$) δ (ppm): 183.0 (C), 157.7 (C), 150.4 (C), 145.3 (C), 138.3 (CH), 124.6 (CH), 123.5 (CH), 117.5 (C), 111.5 (CH), 84.6 (d, $^4J_{C-F}$ = 3.8 Hz, C), 81.7 (d, $^1J_{C-F}$ = 164 Hz, CH_2), 50.6 (d, $^2J_{C-F}$ = 20 Hz, CH_2), 35.7 (CH_2)
m.p.: 165-168 °C
HRMS (ESI): $[M+H]^+$ Calcd. m/z 400.9911, found m/z 400.9910

1-((1-(2-fluoroethyl)-5-iodo-1H-1,2,3-triazol-4-yl)methyl)indoline-2,3-dione (**4**)
(^{1}H NMR, DMSO-d_6, 600 MHz)

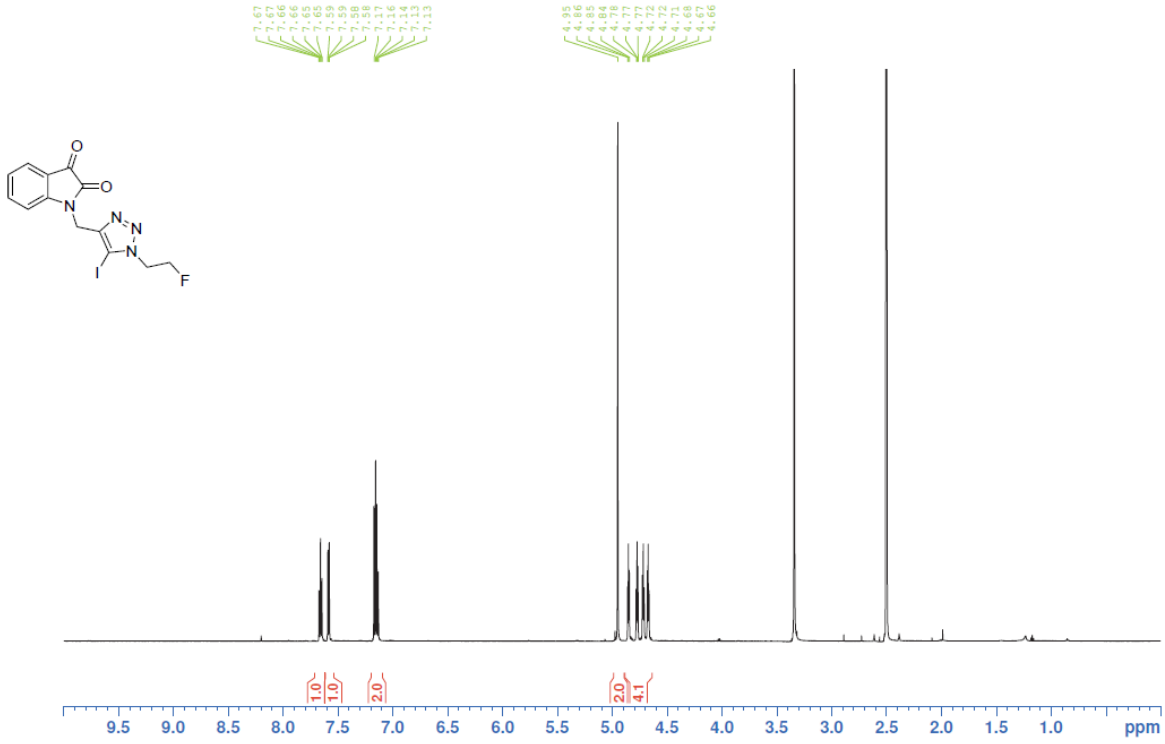

1-((1-(2-fluoroethyl)-5-iodo-1H-1,2,3-triazol-4-yl)methyl)indoline-2,3-dione (**4**)
(^{13}C NMR, DMSO-d_6, 150 MHz)

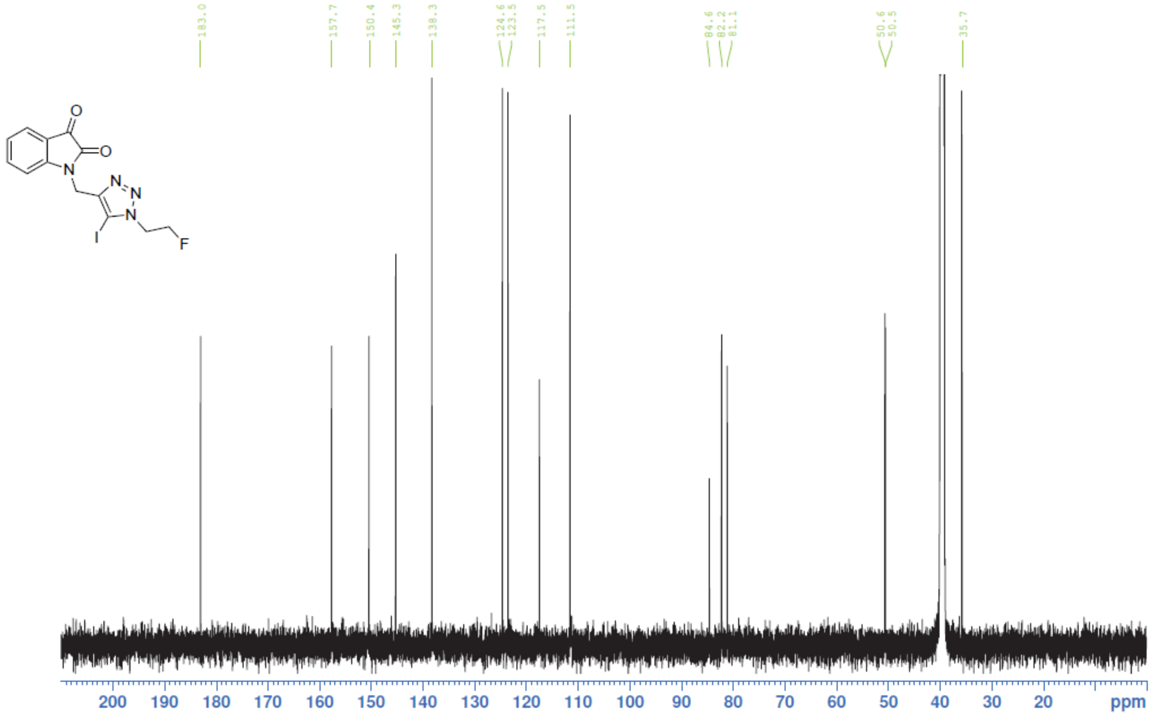

2. Radiolabeling experiments

2.1. Definitions and general considerations

All labeling reactions were performed manually using radioiodine starting materials [^{125}I]NaI and [^{123}I]NaI purchased from Perkin Elmer LAS (UK) Ltd (#NEZ033A020MC), and GE Healthcare Ltd (#CY19V-185MBQ), respectively. Radio-HPLC were performed with an Agilent 1200 HPLC system equipped with a 1200 Series Diode Array Detector and a GABI Star NaI(Tl) scintillation detector (energy window 40-100 keV). The system was used for purification as well as characterization of radiotracers. Columns and conditions used for purification and quality controls (QC) are indicated in the protocol or next to the corresponding chromatogram.

Radiochemical yields were calculated as follows:

- Analytical radiochemical yields (ARCY) were determined using radio-HPLC chromatograms of the quenched crude labeling mixture and refer to the area under the curve (AUC) of the radioactive peak of interest divided by the summed AUC of all other radioactive peaks (radioiodide and potential side-products).
- Isolated radiochemical yields (RCY) refer to the activity of the pure tracer isolated after HPLC divided by the initial activity of [$^{123/125}$I]NaI used for the labeling; no corrections were made for losses during transfer, cartridges trapping and release.

2.2. Cartridge conditioning

Sep-Pak tC18 Plus Light Cartridge (145 mg, Waters Cat. no. WAT036805) was flushed with ethanol (5 mL), HPLC water (10 mL) and air-dried (10 mL).

2.3. Radiolabeling of [$^{123/125}$I]FITI

To a suspension of copper(II) chloride (134 µg, 1.0 µmol) in anhydrous acetonitrile (20 µL) and triethylamine (151 µg, 1.5 µmol) was added a solution of bathophenanthroline (33 µg, 0.1 µmol) in anhydrous acetonitrile (20 µL). A solution of the isatin alkyne **5** (460 µg, 1.0 µmol), in anhydrous acetonitrile (20 µL) was subsequently added and the mixture was left for 5 min at room temperature.

(NB: The isatin alkyne/$CuCl_2$/bathophenanthroline/triethylamine complex in acetonitrile must be freshly prepared and used immediately. Otherwise, the reaction will not work, or give low RCY.)

The resulting solution was added to aqueous [^{125}I]NaI (1-37 MBq, 6.0 µL) in a polypropylene (PP) centrifuge tube (1.5 mL) immediately followed by a stock solution of 2-fluoroethyl azide in anhydrous acetonitrile (40 mM, 25 µL, 1 µmol). The tube was kept at room temperature with occasional agitation for 60 min. The reaction mixture was diluted with a solution of acetonitrile and water (0.5 mL, 3:2 v/v). The resulting solution was purified by HPLC on a ZORBAX® column (300SB-C18, 5 µm, 9.4×250 mm, Agilent) using water and methanol, each containing 0.1% TFA (gradient elution with a flow rate of 3 mL/min, from 40% to 55% methanol content over 50 min, then from 55% to 90% methanol content over 5 min; Figure **3**, $R_t \approx 54$ min). The collected fraction was diluted with water (5 mL), and loaded on a conditioned Sep-Pak tC18 Plus Light Cartridge. After washing with water (5 mL) and drying with air (15 mL), the cartridge was eluted with EtOH (0.5 mL). The product was collected in fractions (0.1 mL). The fractions containing >90% of radioactivity were diluted with saline to give an EtOH content of <10 % (v/v). The final sample was obtained with a radiochemical yield of 55 ±12 % (n = 8) and a radiochemical purity of >99 %.

The iodine-123 labelled analog was obtained using an otherwise identical procedure with the exception that aqueous [^{123}I]NaI (6 µL, 48-132 MBq) was used. The target compound [^{123}I]FITI was obtained in a decay-corrected radiochemical yield of 51% (n = 2), and with a radiochemical purity >99%.

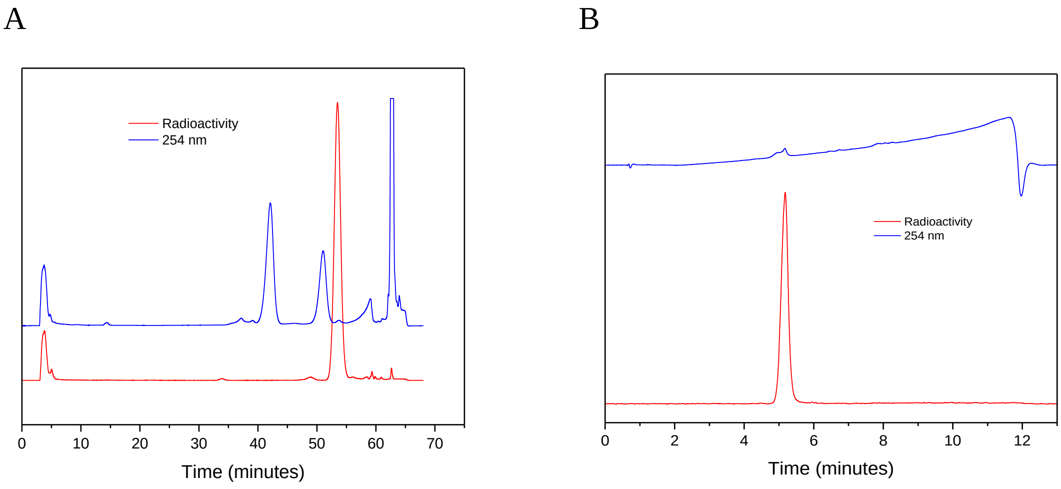

Figure S1. Preparative HPLC profile of a [^{123}I]FITI preparation (A) and quality control of formulated product (B).

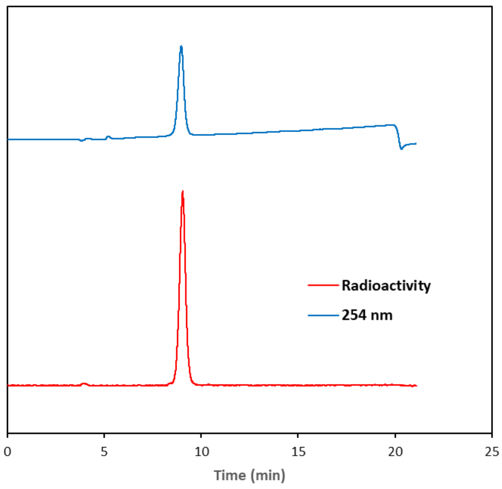

Column: Zorbax RP-18 (300 × 9.4 mm)
Flowrate: 3.00 mL/min

Gradient

Time (min)	H_2O + 0.1% TFA (%)	MeOH + 0.1% TFA (%)
0	40	60
15	10	90

Figure S2. HPLC chromatogram of the isolated radiochemical product [^{123}I]FITI, co-injected with the non-radioactive FITI reference

2.4. Radiolabeling of [^{125}I]4

A suspension of copper(II) chloride (134 µg, 1.0 µmol) and triethylamine (0.21 µL, 1.5 µmol) in anhydrous MeCN (20 µL) was mixed with bathophenanthroline (33 µg, 0.1 µmol) dissolved in anhydrous MeCN (20 µL) and added to a stock solution of alkyne **3** in acetonitrile (50 mM, 20 µL, 1 µmol). After 5 min at room temperature, this mixture was added to aqueous [^{125}I]NaI (8 MBq, 6 µL) in a PP centrifuge tube (1.5 mL). Immediately afterwards, a stock solution of 2-fluoroethylazide in acetonitrile (40 mM, 25 µL, 1 µmol) was added. The reaction mixture was left at room temperature with occasional agitation for 1 h. The reaction mixture was diluted with HPLC mobile phase (0.5 mL 10% MeOH in H_2O + 0.1% TFA v/v/v) and purified by HPLC on a ZORBAX® column (300SB-C18, 5 µm, 9.4×250 mm, Agilent) using water and methanol, each containing 0.1% TFA (gradient elution with a flow rate of 3 mL/min, from 10% to 90% methanol content over 20 min; Figure **S3**, Rt ≈ 13.5 min). The collected fraction was diluted with water (15 mL), loaded on a conditioned Sep-Pak tC18 Plus Light Cartridge, washed with water (5 mL), dried (10 mL air), eluted with EtOH (0.5 mL, 0.1 mL fractions), and the fractions containing >90% of radioactivity

combined and diluted with saline (0.9%, 10% v/v). The final sample was obtained with a radiochemical yield of 39 % and a radiochemical purity of >99%.

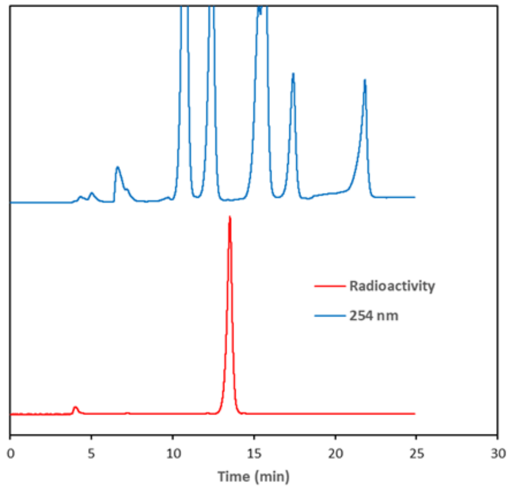

Figure S3. Preparative HPLC chromatogram of [^{125}I]**4**

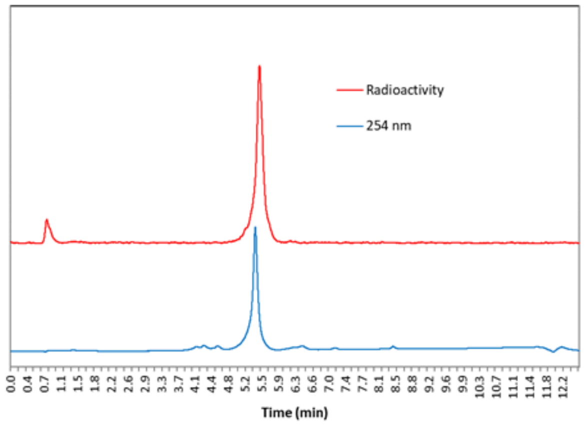

Column: Chromolith® Performance RP-18 (100 × 4.6 mm)
Flowrate: 3.00 mL/min

Gradient

Time (min)	H_2O + 0.1% TFA (%)	MeOH + 0.1% TFA (%)
0	90	10
10	10	90

Figure S3. HPLC chromatogram of the isolated radiochemical product [$^{123/125}$I]**4**, co-injected with the non-radioactive reference compound **4**.

2.5. Log D Measurement

A solution of [^{125}I]FITI or [^{125}I]**4** (0.04 MBq, 10 µL, formulated in saline/10 % EtOH v/v) was added to a mixture of *n*-octanol [0.5 mL, saturated with phosphate buffered saline (PBS, Sigma-Aldrich Cat. P4417)] and PBS (0.5 mL) in a PP vial (1.5 mL). After vortexing for 0.5 min, the vial was spun on a micro-centrifuge (90 s, 10,000 rpm). Aliquots (50 µL) were taken both from the organic and aqueous layers and counted in a gamma well counter. The vortexing, centrifugation and aliquoting steps were repeated four times to reach equilibrium. The full protocol was repeated twice to yield three averages of five organic/aqueous phase measurements.

3. Biology

3.1. Caspase enzyme inhibition assay

Recombinant human caspase-3 and caspase-8 enzymes, substrates Ac-DEVD-AMC and Ac-IETD-AMC, and inhibitors Ac-DEVD-CHO and Ac-IETD-CHO were purchased from Enzo Life Sciences AG. The assay

buffer was consisted of HEPES (pH 7.4, 20 mM), sucrose (10%), NaCl (100 mM), CHAPS (0.1%), and EDTA (2 mM).[1] A Varioskan-LUX (ThermoFisher UK) fluorescence plate reader was used to read 96-well plates in triplicate at 37 ^oC incubation temperature (excitation wavelength = 355 nm, emission wavelength = 460 nm). The inhibitor test compounds were serially diluted in DMSO (1 mM, 0.1 mM, 10 µM, 1 µM, 0.1 µM, 10 nM, 1 nM, and 0.1 pM). The caspase enzyme stock solutions were made up to give 1 U per well. The substrates were prepared in assay buffer solution (40 µM). Inhibitor solution (5 µL) was added to wells containing assay buffer (20 µL) followed by substrate solution (50 µL). After an incubation period at 37 ^oC for 10 min, the enzyme solution was added (25 µL). The wells of the blank experiments contained assay buffer (50 µL) and substrate solution (50 µL). The control wells used assay buffer (20 µL), enzyme solution (25 µL), substrate (50 µL), and DMSO (5 µL). Fluorescence signals were measured once per minute for one hour. The IC_{50} values were computed based on the linear ranges of the dynamic signal profiles using non-linear correlation software (OriginPro 2015). The K_i values were calculated from an average of three IC_{50} measurements using the Cheng-Prusoff equation[2] and a K_m for Ac-DEVD-AMC of 9.7 µM.[3]

3.2. Plasma metabolite analysis

Blood (0.7-1.0 mL) was removed from BALB/c mice shortly after the animals were euthanized and was spun down in a micro-centrifuge at 13,000 rpm for 3 min. The supernatant was decanted and mixed with ice-cold MeCN (1.0 mL) followed by vortexing (1 min) and centrifugation (13,000 rpm, 3 min). The supernatant was diluted with water (1.0 mL) and analyzed by HPLC on a ZORBAX® column (300SB-C18, 5 µm, 9.4×250 mm, Agilent) using water and methanol, each containing 0.1% TFA (gradient elution with a flow rate of 3 mL/min, hold 1 min at 10% methanol content and then from 10% to 90% methanol content over 20 min).

3.3. Biodistribution studies in mice

Biodistribution studies in tumour-bearing mice were carried out using female NOD scid-gamma (NSG) mice (6-8 weeks old, 20-25g) bred in the UCL animal facility. For all studies mice were acclimatized for a week prior to initiation of studies. Mice were housed under sterile conditions in individually ventilated cages, fed with standard chow diet and water *ad libitum* and maintained on an automatic 12 h light cycle at 22–24°C. All animal experiments were performed in accordance with the UK Home Office Animals Scientific Procedures Act 1986 and United Kingdom Co-ordinating Committee on Cancer Research Guidelines for the Welfare and Use of Animals in Cancer Research, and were approved by the University College London Animal Ethics Committee.[4]

3.4. Tumour Models

Subcutaneous tumours were established in female NSG mice using human colorectal adenocarcinoma cell line SW1222 cells. Briefly, 5×10^6 cells were injected into the right flank of mice and allowed to grow to a volume of 0.3-0.5cm^3. Tumour volumes were calliper-measured and calculated using the formula: volume $= 4\pi/3$ (1/2 length × 1/2 width × 1/2 height). Tumours were sized-matched and animals were either untreated or treated with a single dose of clinically formulated etoposide 24 h before radiotracer injection (Eposin, 50mg/kg, i.p.).

3.5. Biodistribution of [^{125}I]FITI in SW1222 tumour-bearing mice

For all biodistribution studies, mice were injected intravenously via the tail vein with 1 MBq of [^{125}I]FITI. Animals were euthanized 60 min after radiotracer injection after which tumours and other selected organs were rapidly excised and weighed. Tissue radioactivity was measured on a gamma counter (Wizard 2470, Perkin Elmer) and uptake of [^{125}I]FITI was calculated as percentage of the injected activity per gram of tissue (%ID/g).

3.6. SPECT-CT imaging of [^{123}I]FITI in SW1222 tumour-bearing mice

The mice were anesthetised by inhalation of 2% isoflurane in oxygen and imaged with a NanoSPECT/CT small animal imager (Mediso Imaging Systems, Budapest, Hungary) for 2 h using a multi-pinhole (nine pinholes, aperture 1.0 mm collimator and a transaxial FOV of 62 mm). Single photon emission computed tomography images were reconstructed using Hi SPECT software and a dedicated ordered subset-expectation maximisation algorithm (Scivis, Goettingen, Germany).

3.7. Statistical analysis

Data are expressed as mean ±SD and comparison between 2 datasets was determined using the Student t test (Prism v6.0 software, GraphPad). Results were considered as statistically significant at a *p*-value of ≤0.05.

References

1. Smith, G. *et al.* Design, synthesis, and biological characterization of a caspase 3/7 selective isatin labeled with 2-[^{18}F]fluoroethylazide. *J. Med. Chem.* **51**, 8057-8067 (2008).
2. Cheng, Y.-C. & Prusoff, W. H. Relationship between Inhibition Constant (K1) and Concentration of Inhibitor Which Causes 50 Per Cent Inhibition (I50) of an Enzymatic-Reaction. *Biochem. Pharmacol.* **22**, 3099-3108 (1973).
3. Nicholson, D. W. *et al.* Identification and inhibition of the ICE/CED-3 protease necessary for mammalian apoptosis. *Nature* **376**, 37-43 (1995).
4. Workman, P. *et al.* Guidelines for the welfare and use of animals in cancer research. *Br. J. Cancer* **102**, 1555-1577, doi:10.1038/sj.bjc.6605642 (2010).
